# A study about factors influencing rice palatability based on changes in sensory and physicochemical properties under different postharvest conditions

**DOI:** 10.1016/j.crfs.2023.100625

**Published:** 2023-10-31

**Authors:** Ah-Na Kim, Oui Woung Kim, Hoon Kim

**Affiliations:** Research Group of Safety Distribution, Korea Food Research Institute, Wanju-gun, 55365, Republic of Korea

**Keywords:** Postharvest management, Rice, Sensory quality, Germination rate, Storage

## Abstract

Postharvest management plays a key role in determining the end-use quality of rice; therefore, a practical approach to inhibit quality deterioration is necessary. In this study, the effects of postharvest management—drying delay time (DDT) and moisture content after drying (DM) immediately after harvesting, and storage temperatures (ST) and periods (SP) of dried paddy rice—on the physicochemical, quality, and sensory properties of rice were comprehensively analyzed. Germination rate, seed viability, fat acidity, and sensory quality tended to significantly deteriorate with increasing DDT, DM, ST, and SP. The highest correlation (*r* = 0.8289) was observed between germination rate and sensory quality, indicating that germination rate can reliably predict sensory quality. Degradation of germination rate and overall sensory quality were analyzed: sensory quality exhibited a more gradual change than germination rate. Lastly, the effects of the postharvest conditions on overall sensory quality were predicted using a regression equation model. DDT, DM, and ST exhibited different patterns of change, which can be used to predict the sensory quality during storage. Germination rate was successfully applied as an influencing factor for the development of a rice-palatability prediction model. The results of this study are useful for maintaining fresh-rice palatability by preventing aging during postharvest storage.

## Highlights

• Effects of postharvest conditions on physicochemical and sensory properties of rice were analyzed.
• Germination rate, seed viability, fatty acidity, and sensory quality varied greatly.
• Germination rate and sensory quality had the highest correlation (*r* = 0.8289).
• Germination rate can be a reliable factor for predicting sensory quality.
• Degradation types indicated germination rate changed more dramatically than sensory quality.

## Graphical abstract

**Figure:**
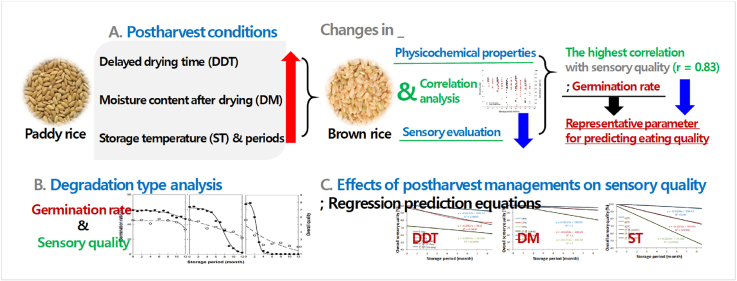

## Abbreviations

CR: cooked rice;
PR: paddy rice,
BR: brown rice,
DDT: drying delay time;
DM: moisture content after drying
ST: storage temperature
SP: storage period
MR: milled rice,
RVA: Rapid viscoanalyzer

## 1 Introduction

Rice (*Oryza sativa* L.) is one of the most widely produced and consumed cereal crops worldwide. It is known as a staple food for over half of the world population, mainly in Asia, Europe, the United States, and Africa (Kubo and Purevdorj, 2004). Rice is served daily as cooked rice (CR), and a small portion is used as an ingredient for processed products. The production and consumption of rice are steadily growing, and are expected to further increase with increasing global population (Kubo and Purevdorj, 2004). To maintain a stable supply, it is essential to increase the productivity and retain the high-quality of rice during its postharvest processes (Qiu et al., 2014). Furthermore, it is necessary to ensure the preservation and availability of rice to address its seasonality (Elert, 2014).

Postharvest management is an integral component in improving and stabilizing the food supply chain before consumer consumption. During the storage of harvested rice, several physicochemical and physiological changes, usually termed as “rice aging,” occur. Thus, postharvest conditions should be carefully controlled to avoid qualitative and quantitative grain loss (Sodhi et al., 2003; Qiu et al., 2014; Genkawa et al., 2008; Tong et al., 2019). Moisture content and temperature are the main factors to control during postharvest treatment because an increase in either accelerates the aging process (Kapoor et al., 2011; Whitehouse et al., 2018). To decrease the moisture content of paddy rice (PR), the postharvest drying process is a necessary unit operation; however, delayed and incomplete drying makes it difficult to maintain the fresh quality of rice during storage (Samaddar et al., 2017). The storage temperature and duration are closely related to the physicochemical mechanisms and their deterioration rates (Zhou et al., 2015). Therefore, appropriate postharvest treatment can minimize the deterioration of physicochemical properties, resulting in a high eating quality of rice.

Sensory analysis techniques have been used to evaluate the palatability and end-use quality of rice depending on its postharvest history (Meullenet et al., 1999). The most important criterion for consumer choice of rice is palatability, that is, sensory eating quality (Yanjie et al., 2018). Recently, consumers with improved standards of living prefer purchasing palatable rice even with a relatively higher cost (Ohtsubo and Nakamura, 2017). However, it is difficult to evaluate the sensory properties of rice before pricing because sensory evaluation requires large amounts of human and time resources and samples, involving the cost of performing sensory panel research and variability between human subjects (Bao, 2018). Several researchers have attempted to identify the factors that influence rice palatability by developing instrumental methods and predictive models. However, there are limitations to applying instrumental methods in the rice industry, such as using the Toyo Taste Meter (Toyo Rice Co., Ltd, Japan), electronic tongue (Uyen et al., 2004), Mido Meter (Toyo Rice Co., Ltd, Japan), Tensipresser instrument (Taketomo Electric Incorporated, Japan), and TA. XT2 texture analyzer (Stable Micro Systems, UK), mainly because of the need for CR during testing. Moreover, there are only few predictive models that are used in industry owing to the delicate, non-unified, and time-consuming experimental methods required for the analysis of the influencing factors with a low determination coefficient. Even now, the identification of the factors and development of models available for describing the eating quality of rice remain a challenge (Bao, 2018). Therefore, there has been considerable research on the impact of different postharvest conditions on the sensory qualities of rice (Meullenet et al., 1999; Lyon et al., 1999; Zhou et al., 2002; Kapoor et al., 2011; Kim et al., 2017; Tong et al., 2019); however, these studies could not predict the eating quality of rice (Bao, 2018). Yu et al. (2017) pointed out that further research is essential to investigate the effect of comprehensive postharvest history and processing on physicochemical factors because these steps consequently control the palatability and sensory perception of appearance, taste, texture, and flavor of rice. Thus, it is necessary to systematically analyze the changes in the overall characteristics and sensory quality of PR treated by various postharvest operations and their correlations to predict the end-use quality and palatability of rice.

This study aims to investigate the changes in the physicochemical and sensory properties of PR as a function of the postharvest treatment conditions, namely the drying delay time (DDT), moisture content after drying (DM), storage temperature (ST), and storage period (SP). All quality parameters, such as the physical (moisture content, weight of 1000 seeds, brown rice (BR) recovery, color, and Rapid viscoanalyzer (RVA) parameters), chemical (protein and amylose contents), and quality (fat acidity, germination rate, seed viability, guaiacol oxidation assay, peroxidase activity, and reducing sugar content), of BR and the sensory evaluation of CR were evaluated over a storage duration of one year. The degradation types of the main influencing factors and overall sensory properties were analyzed. Finally, the influence of the postharvest unit operations on the sensory quality was determined and predicted using a regression equation.

## 2 Materials and methods

### 2.1 Rice samples

The rough short-grain rice cultivar Senuri, which is widely grown in South Korea, was used for this study. The samples were harvested in Yesan County (Chungnam Province; 36.7261038′N latitude, 126.8172175′E longitude). Their initial moisture content was 26% (wet basis). Immediately after harvest, PR was introduced and processed in the Korea Rice Processing Complex. A pre-cleaner (IDS-30C, IGSP, Korea) was used to remove foreign materials, such as small sand particles and straw. The PR was subsequently dried. BR was obtained by hulling the dried PR.

### 2.2 Postharvest management and storage condition

Postharvest management was performed in the Korea Rice Processing Complex, which is a mechanized and automated facility that sequentially dries, stores, processes, and packages rice (Lee et al., 2013; Kim and Kim, 2016). The PR was processed as a function of the postharvest treatment conditions, namely DDT and DM, immediately after harvesting, and various ST and SP after drying to diversify its physicochemical and sensory properties. First, PR was treated at 3 DDTs: 0 (immediately after harvesting), 7, and 14 d. Approximately 1000 kg of PR was placed in a conventional ton-bag immediately after harvesting and stored in a warehouse that could shield from the external environment until drying. During storage, the humidity was approximately 54%, and the temperature increased from 18.7 °C at 0 d to 48.1 and 65.4 °C at 7 and 14 d, respectively, owing to rice respiration. The samples treated with various DDTs were dried at 45 °C using a grain circulation dryer (2 ton/batch, Hansung Industry, Korea). Three DM levels were used for each DDT in the study: 12.0, 12.7, and 18.3% on day 0; 12.7, 13.4, and 16.1% at 7 d; and 11.5, 12.5, and 14.9% at 14 d. The treated samples (10 kg) were sealed in a polyethylene film to prevent changes in moisture content. The packed samples were then stored at different STs of 10, 20, and 30 °C for an SP of one year.

A total of 27 treatment groups were tested in the study (Table 1). Immediately after each SP, BR was produced by removing the husk of the PR using a rubber roll-type husker (THU, Satake, Japan). The changes in the physicochemical properties and quality of the BR were evaluated monthly. These properties included the physical characteristics (moisture content, weight of 1000 seeds, BR recovery, color, and RVA parameters), chemical properties (protein and amylose contents), and quality (fat acidity, germination rate, seed viability, guaiacol oxidation assay, peroxide activity, and reducing sugar content). Finally, rice palatability as a function of the postharvest treatment conditions was analyzed by the sensory evaluation of CR for 8 h every two months.

**Table 1:** Group names of paddy rice treated as a function of dry delay time, moisture content after drying, and storage temperature.

Group names	Drying delay time (DDT, days)	Moisture content after drying (DM, %, w.b.)	Storage temperature (ST, °C)
DDT0-DM18.3-ST10	0	18.3	10
DDT0-DM18.3-ST20			20
DDT0-DM18.3-ST30			30
DDT0-DM12.7-ST10		12.7	10
DDT0-DM12.7-ST20			20
DDT0-DM12.7-ST30			30
DDT0-DM12.0-ST10		12.0	10
DDT0-DM12.0-ST20			20
DDT0-DM12.0-ST30			30
DDT7-DM16.1-ST10	7	16.1	10
DDT7-DM16.1-ST20			20
DDT7-DM16.1-ST30			30
DDT7-DM13.4-ST10		13.4	10
DDT7-DM13.4-ST20			20
DDT7-DM13.4-ST30			30
DDT7-DM12.7-ST10		12.7	10
DDT7-DM12.7-ST20			20
DDT7-DM12.7-ST30			30
DDT14-DM14.9-ST10	14	14.9	10
DDT14-DM14.9-ST20			20
DDT14-DM14.9-ST30			30
DDT14-DM12.5-ST10		12.5	10
DDT14-DM12.5-ST20			20
DDT14-DM12.5-ST30			30
DDT14-DM11.5-ST10		11.5	10
DDT14-DM11.5-ST20			20
DDT14-DM11.5-ST30			30

### 2.3 Physicochemical properties

The BR recovery was calculated as the ratio of hulled PR that was not screened by a string sieve with a wire width of 1.6 mm. The moisture content and weight of 1000 seeds were measured by the American Association of Cereal Chemists (AACC) method 44-15 A (AACC, 2000), in accordance with the method of Bodi et al. (2006). The whiteness and color values were determined using a whiteness tester (C-300–3, Kett Electric Laboratory, Tokyo, Japan) and colorimeter (CM-2500 d, Konica Minolta Sensing, Inc., Japan), respectively. The color values included the L value ranges from dark to white, a-value ranges from green to red, and b value ranges from blue to yellow. The RVA parameters, namely peak viscosity, trough, breakdown, consistency, setback, and peak time, were obtained using a Rapid viscoanalyzer (RVA super 4, Newport Scientific, Sydney, Australia), as described by the AACC method 76-21 (AACC, 2000). The obtained data were processed using the Thermocline window software. The protein and amylose contents were analyzed using a grain analyzer (Infratec 1241, Foss Techator, Hogenas, Sweden) that uses near-infrared transmittance technology.

### 2.4 Quality properties

Fat acidity was determined using the AACC method 02-01 A (AACC 2000). The germination rate was determined as the percentage of seeds that germinated after incubation at 20 °C for 7 d. The tetrazolium test (seed viability), guaiacol oxidation assay, peroxidase activity, and reducing sugar content were measured as described in AOSA (2007), Doerge et al. (1997), Chen and Chen (2003), and Miller (1959), respectively, with slight modifications.

### 2.5 Sensory evaluation

CR samples with different DDT, DM, ST, and SP conditions were prepared for the sensory analysis. First, BR was polished using a Yamamoto rice polisher (friction type, VP-31 T, Yamamoto Co., Tendou, Japan) to obtain the optimum quality of milled rice (MR). In our previous study, the optimum whiteness and milling degree of short grains were 40–41 and 8.9–9.2%, respectively (Kim et al., 2005). The non-defective MR grains, which were selected by a rice sorter (FGS-1000, Satake, Japan), were cooked as described by Kim et al. (2000). MR (800 g) was rinsed and drained twice using a rice cleaner (PR-7J, Aiho, Tokyo, Japan), and was subsequently added to deionized water of a volume that was 1.4 times that of MR (14% moisture basis). MR was cooked using a rice cooker (SJ-185 R rice cooker, Samsung Electric. Co., Suwon, Korea) for 30 min, and rested for 10 min after cooking. The rice in the middle, i.e., except for that within 1 cm from the side and bottom of the pot, was transferred to a bowl (23 cm diameter × 12 cm depth) for analysis. CR was carefully mixed five times with an up–down motion using a fork (length 35 cm) and cooled at 20 °C for 5 min. After repeating the mixing and cooling twice, CR (50 g) was portioned with a stainless-steel ice cream scoop (diameter 6 cm, Saejang Co., Seoul, Korea) into lidded white bowls (diameter 8.5 cm × depth 5 cm) and labeled with random three digit codes. The samples were served to trained panelists at room temperature at random.

Preferred eating quality depends on the geographical region and culture. Asia, which is the largest continent and contains all major rice-producing countries, has different preferences on rice. Northeast Asians prefer the soft, sticky, and lumpy texture of freshly harvested rice, whereas South Asians like the fluffy, non-sticky, and almost free-flowing texture of aged rice (Bhattacharya, 2011). This study considered freshly harvested rice in order to have the desired palatability according to the preference of Northeast Asians. The sensory evaluation of CR was performed following the methods of Kim et al. (2000, 2017). The panelists from the Korea Food Research Institute (Seongnam-si, Korea) participated in the sensory analysis every 2 months for a year. Thirty-two out of 52 panelists were selected based on panel performance as in Cross et al. (1978). The panel included 32 assessors (19 women and 13 men) between 20 and 36 years of age and with a high level of education and previous experience in sensory analysis. All experiments followed the relevant guidelines and regulations according to the Institutional Review Board of Korea National Institute for Bioethics Policy (KoNIBP). All participants had provided informed consent and were trained well for 10 d in 30 min/day sessions. In the training session, CRs of various eating qualities were presented, and the participants were trained with a guide for evaluating the degree of quality (Kim et al., 2000). The sensory evaluation took place in a test room in accordance with ISO 8589 (2007). Most panelists agreed on the high and low quality of CR in terms of odor, appearance, texture, and taste. Thus, the final panel group without the outliers rated the CR for the intensities of individual attributes, namely appearance, odor, taste, texture, and overall quality, on a 9-point structured scale (1 = very low and 9 = very high) in duplicate.

### 2.6 Statistical analysis

Each treatment group was tested by storing one bag at a time. All measurements were performed in triplicate, and the results are presented as mean values. The significant differences between the characteristics of each sample were analyzed by analysis of variance using a statistical analysis system (ver. 9.0, SAS, Inc., Cary, NC, USA) using a Duncan's multiple range test (p < 0.05). For the characteristics that were statistically different between the samples, the mean values were compared using the Student–Newman–Keuls (SNK) multiple comparisons test (p < 0.05). In addition, the effects of the postharvest conditions, namely DDT, DM, and ST, on the deterioration of rice palatability were predicted using a regression equation; an initial fresh palatability value of 100% was set.

## 3 Results and discussion

### 3.1 Changes in the physicochemical properties

**Fig. 1:**
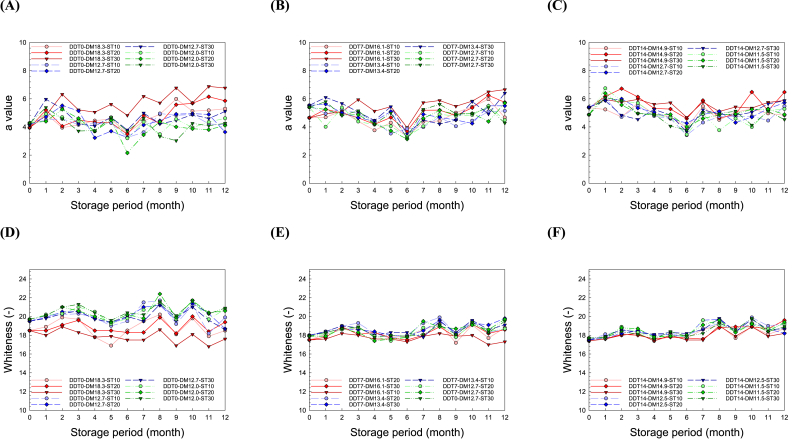
Effects of the postharvest treatment conditions, namely delay times before drying (DDT) and moisture contents after drying (DM), and storage condition after drying, namely storage temperatures (ST) and period (SP), on (A, B, C) a-value and (D, E, F) whiteness of rice for DDT0, DDT7, and DDT14, respectively.

The physical properties, namely moisture content (Fig. S1A–C), weight of 1000 seeds (Fig. S1D–F), BR recovery (Figs. S1G and H), color (Fig. 1 and S2), and RVA parameters (Fig. S3), of the hulled BR were analyzed. The physical parameters exhibited substantial differences among the groups treated with different DDTs, DMs, STs, and SPs. The moisture content of the DDT0-DM18.3-ST30, DDT7-DM16.1-ST30, and DDT14-DM14.9-ST30 groups, which have DM and ST values greater than 14.9% and 30 °C, respectively, gradually decreased during storage in accordance with the result of the research conducted by Park et al. (2012). Meanwhile, only slight changes in the moisture content were noted for the other groups. The weight of 1000 seeds of all groups was detected ranged from 22.47 to 24.45. There were minimal changes in the weight of 1000 seeds for all groups without significant difference (p > 0.05). The BR recovery slightly increased with DM; nonetheless, the differences were negligible (p > 0.05).

For the color values, the L and b values did not differ under different postharvest processes (Fig. S2). The DDT0-DM18-ST30 group, which has the highest moisture content, showed a gradual increase in the a-value and decrease in whiteness with SP, unlike the other groups (Fig. 1). Furthermore, prolonged DDT decreased the whiteness in all groups, regardless of the DM and ST conditions. Several studies have reported that elevated moisture content, water activity, temperature, respiration rate, SP, and carbon dioxide content among other factors may cause yellowing of rice (Adams and Rinne, 1980; Dillahunty et al., 2000; Kapoor et al., 2011; Whitehouse et al., 2018). There were significant changes in the breakdown and consistency of the RVA parameters (Fig. S3). The RVA parameters for all groups increased with the DDT, DM, ST, and SP. The DDT0-DM18-ST30 group, which has the highest level of moisture, exhibited the most dramatic increase. For the chemical properties, the protein and amylose content of BR did not exhibit any significant tendency under different DDT, DM, ST, and SP conditions (Fig. S4). Overall, DM was the most influential factor among physicochemical properties of rice as a function of different postharvest management. Excessive moisture after drying can cause physical damage by increasing sensitivity to improper postharvest history.

### 3.2 Changes in quality

**Fig. 2:**
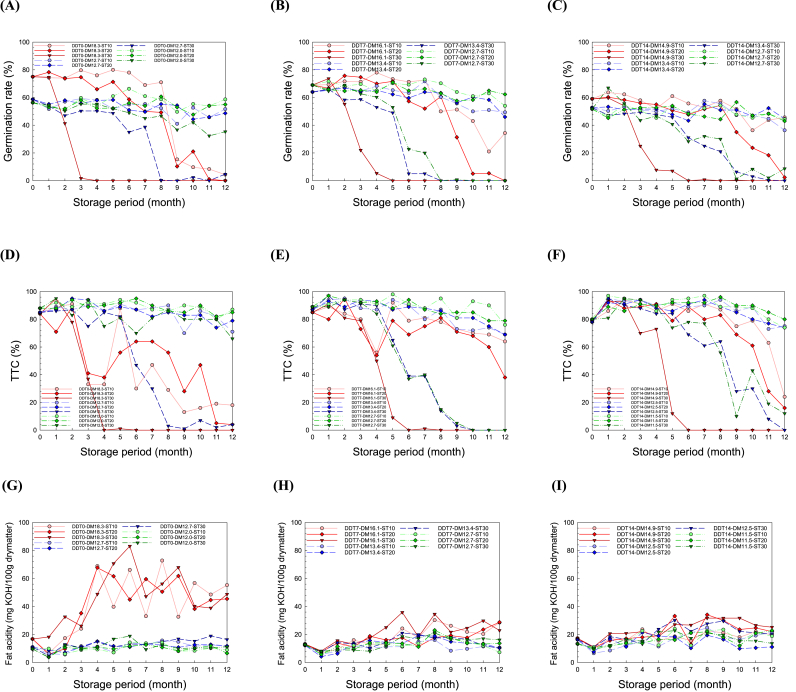
Effects of postharvest process conditions, namely delay times before drying (DDT) and moisture contents after drying (DM), and storage conditions after drying, namely storage temperatures (ST) and period (SP), on the (A, B, C) germination rate, (D, E, F) seed viability (TTC), and (G, H, I) fat acidity for DDT0, DDT7, and DDT14, respectively.

The effects of postharvest management on the quality of BR, namely the germination rate, seed viability, fat acidity, guaiacol oxidation, peroxidase activity, and reducing sugar content, were evaluated. The largest change was found in the rice germination rate as a function of the postharvest treatments (DDT and DM) and storage conditions (ST and SP) (Fig. 2A–C). Particularly, the increases in DDT, DM, ST, and SP deteriorated the germination rate. Seed germination, which is essential for next-generation plant growth, is a physiological process that begins with dry mature seed imbibition and ends with radicle protrusion (Bewley, 1997; Bewley et al., 2013). The seeds successfully germinate under appropriate environmental conditions, such as water content, temperature, atmosphere, and light (Holdsworth et al., 2008).

The DDT0-DM12.0-ST30 samples maintained a higher germination rate than the DDT14-DM11.5-ST30 samples during storage, even though they contained higher moisture content than the latter. This indicates that the germination rate improves as the DDT decreases. Moisture plays a key role in seed physiology. Tirawanichakul et al. (2004) reported that harvested PR with the average moisture content of 20–25% wet basis should be dried immediately until it reaches the optimum moisture content. With delayed drying, rice respiration continues to generate heat, water, and CO_2_ from the oxidation of sugars, resulting in quality deterioration and nutrition consumption (Dillahunty et al., 2000; Kwon et al., 2006). Similarly, He and Yang (2013) reported that seed storage is often accompanied by progressive seed aging and loss of germination vigor, even under optimal storage conditions. The DDT0-DM18 group, which has the highest moisture content, showed the highest germination rate at the beginning of SP; however, this group also showed the most dramatic reduction rate among all groups. These results indicate that DM has a substantial effect on rice germination. Roberts (1961) reported that an increase in moisture content increases the reduction rate of seed viability, namely germination, during storage at the temperatures of 27–47 °C. Delouche (1973) reported that rice seeds with moisture content above 18% are more susceptible to heat damage, compared to seeds with lower moisture content. Several researchers recommended the importance of storage of rice at the optimum moisture content of 12–13% wet basis (Tirawanichakul et al., 2004; Genkawa et al., 2008; Atungulu et al., 2015; Janaun et al., 2016). Low moisture content depresses chemical reactions, respiration rate, water activity, and insect damage, which may prevent the reduction in germination rate by regulating metabolic processes (Han and Yang, 2015).

Temperature plays an important role in the survival of rice seeds. The germination rates of all the samples stored at the highest temperature of 30 °C decreased dramatically to 0% after one year. The increase in ST can cause seed viability loss and germination deterioration owing to degenerative metabolism, according to Roberts (1961) and Genkawa et al. (2008). They reported that higher temperatures resulted in a faster reduction in seed germination and viability during storage. Starch and sugar are essential for supplying germination energy by entering the glycolysis pathway and tricarboxylic acid cycle (Yang et al., 2007; Han and Yang, 2015). However, the storage of PR under high temperature and humidity might inhibit sucrose transferase hydrolase, thereby decreasing the starch and sucrose content (Zhao et al., 2017). In addition, a longer SP resulted in lower germination rates in most groups. Tong et al. (2019) reported that prolonged storage leads to structural changes in the cell wall and major chemical components, ultimately resulting in changes in the overall quality induced by the reactions of numerous endogenous enzymes. The failure of seed germination is attributed to the degradation of the mitochondrial membrane, impairment of energy supply mechanisms, and lower rates of biosynthesis essential for germination (Seshu et al., 1988; Gidrol et al., 1998). Therefore, prompt and proper drying and storage are essential for increasing seed germination rate.

Seed viability was analyzed using the tetrazolium test to determine the percentage of seeds that can be germinated based on the activity of dehydrogenase enzymes (Soares et al., 2016). Similar to the germination rate, there was a clear difference on the seed viability of the groups treated at different postharvest and storage conditions (Fig. 2D–F). In particular, the seed viability decreased with increasing DDT, DM, ST, and SP. Seed viability deteriorates over time, even under optimum dry and cold postharvest conditions (Soares et al., 2016).

Fat acidity is used as an indicator of quality deterioration during rice storage because lipid dissolution progresses more rapidly than that of proteins and starch (Genkawa et al., 2008). The increases in the DDT, DM, ST, and SP increased the fat acidity of BR (Fig. 2G–I), which does not agree with the results reported by Park et al. (2012) and Kim et al. (2014). In particular, they reported that the fat acidity of rice tended to increase with increasing ST and SP. The factor with the greatest impact on fat acidity was DM, as demonstrated by the steepest increase in the DDT0-DM18.3 group, which had the highest moisture content. The high moisture content of the grains promotes lipid hydrolysis to produce free fatty acids and induce lipid oxidation to yield hydroperoxides, resulting in a higher fat acidity (Paraginski et al., 2014; Tong et al., 2019).

There was no clear effect on the guaiacol oxidation assay and peroxidase activity as a function of the postharvest operation conditions (Figs. S5A–C and S5D–F). The initial reducing sugar content before storage increased with increasing DDT. However, longer DDT further decreased the reducing sugar content until 2 months (Fig. S5G–I). Therefore, appropriate postharvest processes are necessary to achieve more consistent and desirable quality of rice grains. Furthermore, the germination rate of rice may be considered an indicator of the effect of postharvest management on its physicochemical properties and quality.

### 3.3 Sensory evaluation

**Fig. 3:**
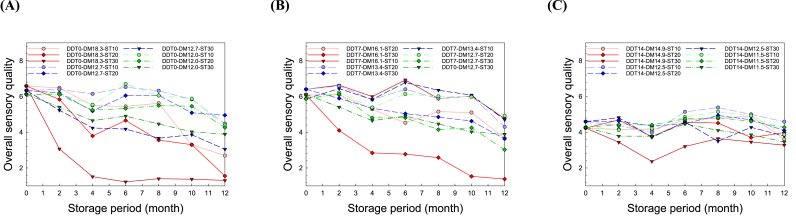
Effects of the postharvest treatments, namely delay times before drying (DDT) and moisture contents after drying (DM), and storage condition after drying, namely storage temperatures (ST) and period (SP), on the overall sensory quality of rice. (A) DDT0, (B) DDT7, and (C) DDT14.

There were obvious differences in the sensory properties of the groups treated with various postharvest and storage conditions (Fig. 3). These changes reflect those of other attributes, such as appearance, odor, taste, and texture, showing similar tendencies. The increase in DDT, DM, ST, and SP resulted in significant deterioration of the sensory quality, similar to the trend for the quality properties. The DDT of 14 d before storage had the greatest effect on the deterioration of the initial sensory properties compared to the other properties. During storage after drying, the increase in DM and ST decreased the sensory properties. After 4 months, the lowest overall quality was observed in the DDT0-DM18.3 group, which has the highest moisture content. Several researchers have reported that the physicochemical properties and quality affected by the postharvest conditions effectively change the sensory quality of rice (Kondō and Okamura, 1937; Shibuya et al., 1974; Meullenet et al., 2000; Lyon et al., 2000; Kim et al., 2000; Zheng and Lan, 2007). In particular, elevated storage moisture content, temperature, and duration are causative factors of rice aging, resulting in an off-odor and a darker, browner color (Barber, 1972; Bhattacharya, 2011; Bao, 2018). Therefore, sensory evaluation is useful for the definitive estimation of the end-use quality as affected by various postharvest processing operations, such as drying and storage (Meilgaard et al., 1991; Meullenet et al., 1999). The sensory evaluation results suggest that optimum postharvest processing of grain could contribute to the breeding of rice with high eating quality.

### 3.4 Degradation of the germination rate and overall sensory quality

Palatability is the most important factor in purchasing rice. Sensory analysis has been used to evaluate the end-use quality. However, this analysis requires numerous trained human participants (Sharif et al., 2017). Further, it is necessary to find an influencing factor that can be representative of sensory quality in the rice industry. In this study, postharvest treatment conditions had the greatest influence on the change in the germination rate among all physicochemical and quality properties. In addition, germination rate has the highest Pearson correlation with the overall sensory quality (*r* = 0.8289). Thus, it is considered an influencing factor that can represent rice palatability with the highest accuracy among all factors. Several literature reviews have reported that seed deterioration reduces seed viability, vigor, and germinability. However, there is yet to be a study on the correlation between germination and eating quality of rice. Most studies have considered chemical properties (moisture, amylose, protein, and mineral content), physical properties (color, RVA parameters, and texture), and fat acidity among others as the main factors influencing sensory quality (Chikubu et al., 1985; Sakurai et al., 1988; Horino and Okamoto, 1992; Ohtsubo et al., 1993; Lu and Zhu, 2014) because they can be directly associated with sensory attributes.

Seed aging is a process that leads to a delayed decrease in the germination rate and total loss of seed viability (Zhang et al., 2016). During aging, the abundance of glycolytic enzymes and two fermentation enzymes, pyruvate decarboxylase and alcohol dehydrogenase, in the embryos increased. This developed hypoxic conditions in the embryos, subsequently causing ethanol accumulation, which reduces seed viability (Zhang et al., 2016). After harvesting, PR is generally stored in a warehouse for more than one year. The aging of rice also causes significant physicochemical and physiological changes (Zhou et al., 2015; Saikrishna et al., 2018; Tong et al., 2019). In particular, aging changes the chemical composition of rice, such as the amounts of starch, lipids, and proteins, and the interactions between them. Lipids are degraded by two possible processes: hydrolysis to produce free fatty acids, or oxidation to produce hydroperoxides and carbonyl compounds (Zhou et al., 2002; Thanathornvarakul et al., 2016; Saikrishna et al., 2018). Hydrolyzed free fatty acids can form complexes with amylose, hydroperoxide, and carbonyl compounds, which can accelerate protein oxidation and condensation as well as accumulation of volatile carbonyl compounds. Volatile compounds, particularly hexanal, contribute to the off-flavor of rice. Aged rice is harder and less sticky than freshly harvested rice owing to protein oxidation, that is, formation of disulfide linkages, other cross-linking reactions in protein molecules, and increased strength of the micelle binding of starch. Thus, it can be concluded that rice aging due to improper postharvest history can deteriorate the germination and eating quality of rice based on the sufficient evidence that germination rate is a good indicator of rice palatability.

**Fig. 4:**
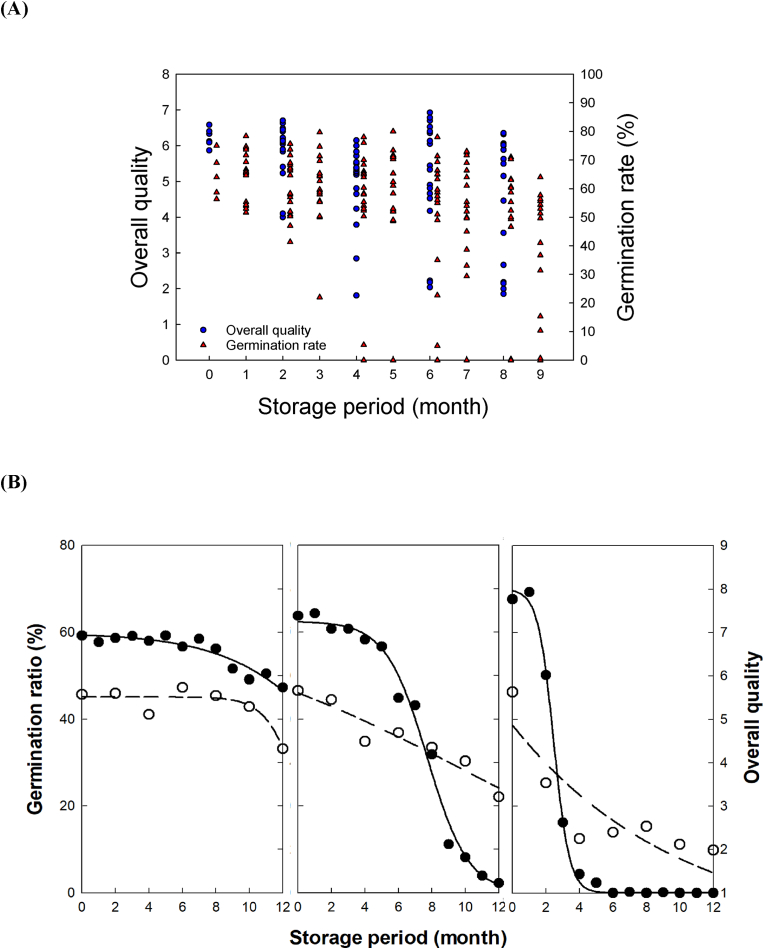
(A) Changes in the germination rate and overall sensory quality of rice as a function of postharvest treatments, namely delay times before drying and moisture contents after drying, and storage condition after drying, namely storage temperatures and period. (B) Change in the overall sensory quality according to the degradation types of the germination rate.

The results of the overall sensory quality and germination are presented together to determine their relationship in Fig. 4A. There are different patterns of change based on the storage conditions. The change patterns were divided into three types according to the degradation patterns of the germination rate (Fig. 4B). For the first type, which has high germination rates, the overall sensory quality is well retained during storage. The second and third types showed curvilinear degradation of the germination rate following an S-curve, whereas the overall sensory quality exhibited a linear decrease. For the second type, the germination rate reached zero at the end of the storage period, and the overall sensory quality slowly decreased with SP. For the third type, the germination rate decreased to zero before half of the SP (one year), which is the highest reduction rate. Meanwhile, the rice samples collected at 0 months after postharvest treatment had an already low overall sensory quality, which gradually decreased to the lowest quality after storage for a year. The analysis of the degradation pattern indicated that the germination rate changed more dramatically, whereas the overall sensory quality varied gradually with linear changes as a function of postharvest conditions.

### 3.5 Effects of postharvest management on rice palatability

The effects of postharvest management, namely DDT, DM, and ST, on rice palatability were predicted using a regression equation model based on the results of the overall sensory quality evaluation. The initial overall sensory quality of the freshest rice was regarded as 100% under the basic conditions of DDT0, DM15, and ST20. The overall sensory qualities of DDT0, DDT7, and DDT14 were 100.00, 99.59, and 71.61%, respectively. Equation (1) presents the initial overall sensory quality (OSQ, %) before storage as a function of DDT (day), as follows:

(1) $$\text{OSQ}=-0.2813x^{2}+1.91\text{DDT}+100\;\left(R^{2}=0.9999\right)$$

**Fig. 5:**
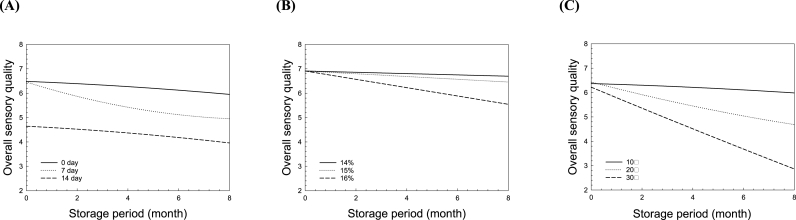
Regression prediction models for the overall sensory quality of rice as a function of storage period and postharvest treatment conditions, namely (A) delay times before drying and (B) moisture contents after drying, and (C) storage condition after drying, namely storage temperatures.

Fig. 5A shows the regression curves of the overall sensory qualities of the rice samples treated with DDTs of 0, 7, and 14 d (Equations (2), (3), (4), respectively) during storage (month) based on the standard of DM15 and ST20:

(2) $$\text{OSQ}=-0.0322\text{SP}+100.57\;\left(R^{2}=0.9898\right)$$

(3) $$\text{OSQ}=-0.0907\text{SP}+96.80\;\left(R^{2}=0.9639\right)$$

(4) $$\text{OSQ}=-0.0413\text{SP}+72.34\;\left(R^{2}=0.9898\right)$$

Equation (1) suggests that there were few changes in the initial overall sensory quality until DDT7. However, an increase in DDT from 7 to 14 d dramatically reduced the quality of rice. During storage after DDT, the reduction rates of the overall sensory quality at DDT0, DDT7, and DDT14 were −1.03, −2.90, and −1.32%/month, respectively. The highest reduction rate was observed for DDT7, where the deterioration of its palatability was not immediately noted after DDT but rather during storage. In contrast, DDT14 exhibited an early deterioration of the initial palatability, resulting in a relatively lower reduction rate than DDT7.

The regression equations of DM14, DM15, and DM16 (Equations (5), (6), (7), respectively) were fitted to the data of the overall sensory quality during storage based on the standard of DDT0 and ST20 (Fig. 5B).

(5) $$\text{OSQ}=-0.0121\text{SP}+100.01\;\left(R^{2}=0.9999\right)$$

(6) $$\text{OSQ}=-0.0256\text{SP}+100.03\;\left(R^{2}=0.9999\right)$$

(7) $$\text{OSQ}=-0.0772\text{SP}+100.08\;\left(R^{2}=0.9999\right)$$

The reduction rates of the overall sensory quality (%/month) of rice treated under DM14, DM15, and DM16 were −0.39, −0.82, and −2.47%/month, respectively. Furthermore, the reduction rates of the overall sensory quality as a function of DM were fitted as follows:

(8) $$\text{Reduction}\;\text{rate}\;\text{of}\;\text{OSQ}=0.6085{\text{DM}}^{2}-17.21\text{DM}+122.09\;\left(R^{2}=0.9999\right)$$

The functional equation indicated a logarithmic dependence on DM; under DMs of 15.0–16.0%, the reduction rates of the overall sensory quality were drastically greater than those under DMs of 14.0–15.0%.

The regression equations for ST10, ST20, and ST30 (Equation (9), (10), (11), respectively) as a function of the overall sensory quality during storage are presented in Fig. 5C.

(9) $$\text{OSQ}=-0.0236\text{SP}+100.41\;\left(R^{2}=0.9900\right)$$

(10) $$\text{OSQ}=-0.1071\text{SP}+99.95\;\left(R^{2}=0.9958\right)$$

(11) $$\text{OSQ}=-0.2059\text{SP}+97.57\;\left(R^{2}=0.9999\right)$$

The reduction rates of the overall sensory quality of rice under ST10, ST20, and ST30 were −0.75, −3.43, and −6.59%/month, respectively. Equation (12), which was obtained by the reduction rates according to different STs, shows a non-logarithmic linear increase with increasing ST, unlike that of DM (Equation (8)).

(12) $$\text{Reduction}\;\text{rate}\;\text{of}\;\text{OSQ}=0.2917{\text{ST}}^{2}-2.2436\text{ST}\;\left(R^{2}=0.9976\right)$$

Consequently, shorter DDT and lower DM and ST resulted in better rice palatability. In addition, the results of the predictive equations indicated that postharvest measurements have different effects on rice palatability. The equations can be used not only for the prediction of changes in the final eating quality of rice during postharvest processing, but also to improve the postharvest conditions.

## 4 Conclusion

A comprehensive analysis of the changes in the physicochemical, quality, and sensory properties of PR, and their interactions as a function of postharvest treatments, namely DDT, DM, ST, and SP, was conducted. Prolonged DDT and high DM, which are related to moisture, caused minimal degradation of several physicochemical properties. However, there were significant differences in the sensory and quality properties, including the germination rate, seed viability, and fat acidity, which deteriorated with increasing DDT, DM, ST, and SP. Thus, the control of temperature and moisture content is the most important factor in maintaining desirable sensory properties and quality during rice storage. Based on the results, the influencing factors representing sensory quality were selected, and their correlation degradation types were discussed. The germination rate had a high correlation (*r* = 0.8289) with the overall sensory quality compared to the other properties. The germination rate of rice seeds can be a representative factor that influences rice palatability. The overall sensory quality exhibited a gradual and constant change, whereas the germination rate showed a more dramatic change. Finally, the effects of the postharvest management and their conditions on the overall sensory quality were predicted using a regression equation model. DDT7 had the highest reduction rate during storage, even with the similar initial sensory quality as that of DDT0. DDT of more than 7 d reduced the initial sensory quality of increase before storage. The reduction rate of the overall sensory quality during storage exhibited a logarithmic increase with DM and linear increase with ST. This study can be applied to monitor changes in the physicochemical and sensory properties of rice to improve the decision-making regarding rice management. Thus, the results of this study can help maintain the palatability of rice by preventing aging during postharvest processing. Further, we successfully developed a rice palatability prediction model with high reliability that can be applied to the rice industry using the germination rate as the influencing factor.

## CRediT authorship contribution statement

**Ah–Na Kim:** Investigation, Visualization, Writing – original draft, preparation. **Oui Woung Kim:** Conceptualization, Methodology, Writing – Reviewing, and Editing. **Hoon Kim:** Conceptualization, Supervision, Writing – Reviewing, and Editing.

## Declaration of competing interest

The authors declare that they have no known competing financial interests or personal relationships that could have appeared to influence the work reported in this paper.

## Data Availability

Data will be made available on request.
