# Supplementary material for: A study about factors influencing rice palatability based on changes in sensory and physicochemical properties under different postharvest conditions
Source: Curr Res Food Sci. 2023 Oct 31;7:100625. doi: 10.1016/j.crfs.2023.100625 (PMC10660032; doi:10.1016/j.crfs.2023.100625)
Supplement: Multimedia component 4 [file mmc4.docx]

**Fig. S4.**

**(A) (B) (C)**

**(D) (E) (F)**

**Fig. S4.** Effects of postharvest process conditions, namely delay times before drying (DDT) and moisture contents after drying (DM), and storage conditions after drying, namely storage temperatures (ST) and period (SP), on the (A, B, C) protein content, (D, E, F) and amylose content of DDT0, DDT7, and DDT14, respectively.
