# Supplementary material for: A study about factors influencing rice palatability based on changes in sensory and physicochemical properties under different postharvest conditions
Source: Curr Res Food Sci. 2023 Oct 31;7:100625. doi: 10.1016/j.crfs.2023.100625 (PMC10660032; doi:10.1016/j.crfs.2023.100625)
Supplement: Multimedia component 7 [file mmc7.docx]

**Table S1.** Effects of postharvest treatments such as different delay times before drying (DDT) and moisture contents after drying (DM) on initial sensory evaluation of rice.

| DDT (days) | DM (%) | Sensory properties | | | | |
| --- | --- | --- | --- | --- | --- | --- |
|  |  | Appearance | Odor | Taste | Texture | Overall sensory quality |
| 0 | 18.3 | 7.13^a^ | 6.67^a^ | 6.56^a^ | 6.13^a^ | 6.57^a^ |
|  | 12.7 | 6.87^a^ | 6.76^a^ | 6.34^a^ | 6.02^a^ | 6.32^a^ |
|  | 12.0 | 6.53^a^ | 6.53^a^ | 6.34^a^ | 5.87^a^ | 6.11^a^ |
| 7 | 16.1 | 6.84^a^ | 6.21^a^ | 6.23^a^ | 6.13^a^ | 6.08^a^ |
|  | 13.4 | 6.88^a^ | 6.61^a^ | 6.58^a^ | 6.15^a^ | 6.41^a^ |
|  | 12.7 | 6.30^a^ | 6.30^a^ | 6.05^a^ | 5.68^ab^ | 5.87^a^ |
| 14 | 14.9 | 5.68^b^ | 4.08^b^ | 4.38^b^ | 4.95^b^ | 4.23^b^ |
|  | 12.5 | 5.56^b^ | 4.39^b^ | 4.64^b^ | 5.03^b^ | 4.59^b^ |
|  | 11.5 | 5.37^b^ | 4.20^b^ | 4.41^b^ | 4.88^b^ | 4.27^b^ |
